# The early molecular events leading to COFILIN phosphorylation during mouse sperm capacitation are essential for acrosomal exocytosis

**DOI:** 10.1016/j.jbc.2022.101988

**Published:** 2022-04-26

**Authors:** Liza J. Schiavi-Ehrenhaus, Ana Romarowski, Martina Jabloñski, Darío Krapf, Guillermina M. Luque, Mariano G. Buffone

**Affiliations:** 1 Instituto de Biología y Medicina Experimental, Consejo Nacional de Investigaciones Científicas y Técnicas (IBYME-CONICET), Buenos Aires, Argentina; 2 Department of Veterinary and Animal Science, University of Massachusetts, Amherst, Massachusetts, USA; 3 Instituto de Biología Molecular y Celular de Rosario (IBR), Consejo Nacional de Investigaciones Científicas y Técnicas (CONICET), Universidad Nacional de Rosario (UNR), Rosario, Argentina

**Keywords:** small GTPases, SSH1, PAK4, LIMK1, actin, ADF, actin-depolymerizing factor, AR, acrosomal exocytosis, BSA, bovine serum albumin, CsA, cyclosporin A, FITC, fluorescein isothiocyanate, HRP, horseradish peroxidase, LIMK1, LIM-kinase 1, OA, okadaic acid, PAKs, p21-activated kinases, pLIMK1, phosphorylated LIMK1, PNA, peanut agglutinin lectin, ROCKs, Rho-associated activated kinases, SSHs, Slingshot family of protein phosphatases, SeA, sennoside A

## Abstract

The actin cytoskeleton reorganization during sperm capacitation is essential for the occurrence of acrosomal exocytosis (AR) in several mammalian species. Here, we demonstrate that in mouse sperm, within the first minutes of exposure upon capacitating conditions, the activity of RHOA/C and RAC1 is essential for LIMK1 and COFILIN phosphorylation. However, we observed that the signaling pathway involving RAC1 and PAK4 is the main player in controlling actin polymerization in the sperm head necessary for the occurrence of AR. Moreover, we show that the transient phosphorylation of COFILIN is also influenced by the Slingshot family of protein phosphatases (SSH1). The activity of SSH1 is regulated by the dual action of two pathways. On one hand, RHOA/C and RAC1 activity promotes SSH1 phosphorylation (inactivation). On the other hand, the activating dephosphorylation is driven by okadaic acid—sensitive phosphatases. This regulatory mechanism is independent of the commonly observed activating mechanisms involving PP2B and emerges as a new finely tuned modulation that is, so far, exclusively observed in mouse sperm. However, persistent phosphorylation of COFILIN by SSH1 inhibition or okadaic acid did not altered actin polymerization and the AR. Altogether, our results highlight the role of small GTPases in modulating actin dynamics required for AR.

In mammalian species, the male gamete must reside in the female tract for a period of time to acquire its fertilizing ability. To achieve that, sperm undergo a series of biochemical and structural modifications, collectively called capacitation (1, 2). Functionally, capacitation is associated to the acquisition of the ability to undergo the acrosomal exocytosis (also known as acrosome reaction; AR). In the case of mouse AR, its occurrence in the upper segments of the oviduct (3, 4, 5) is required for the appropriate relocalization of proteins involved in sperm–egg fusion (6).

From a molecular point of view, signaling pathways leading to AR are complex and temporarily, they can be divided in two groups: (i) those that take place during capacitation and prepare the cell to respond to the appropriate AR agonist; (ii) those that occur after a given stimuli promoting the initiation of the exocytotic process. Examples of signaling pathways in the first group are the membrane potential hyperpolarization (7), actin polymerization (8), acrosomal pH alkalinization (9) and swelling of the acrosome (10), among others. In the second group, we can highlight the activation of the fusion machinery (11), increase in intracellular Ca^2+^ (12) and dynamic changes of filamentous actin (F-actin) structures present in the sperm head (8, 13, 14, 15). In this group, these signaling pathways become activated by molecules in the female reproductive tract that are not fully established. Thus, the actin cytoskeleton, as it does in other exocytotic events, plays a crucial role in priming the cell for exocytosis and later, once it was triggered, in regulating fusion steps occurring in a timely and orderly manner.

Several studies describe changes in actin dynamics during sperm capacitation in numerous mammalian species (16, 17, 18, 19, 20, 21). We previously showed that actin polymerization/depolymerization in the head of mouse sperm is mainly regulated by RHOA/C and RAC1, two members of the Rho-family of small GTPases (22). Activation of RHOA/C and RAC1 promotes LIM-kinase 1 (LIMK1) phosphorylation and its consequent activation (22). LIMKs are activated through downstream protein kinases of Rho-family GTPases, such as Rho-associated activated kinases (ROCKs) and p21-activated kinases (PAKs) (23, 24). We described that ROCK1, a common downstream effector of RHOA/C, is expressed in mouse sperm and contributes to LIMK1 phosphorylation (22). On the other hand, RAC1-dependent LIMK1 activation is controlled by PAKs, but their expression and function in mouse sperm remains unclear. In this regard, it was recently reported that RAC1 inhibition alters capacitation and AR and specifically regulate F-actin formation in the apical region of the guinea pig acrosome (a commons site of AR initiation) (25).

The actin-depolymerizing factor (ADF)/COFILIN family comprises three proteins that are expressed in mice: ADF (also known as destrin), COFILIN-1, and COFILIN-2. ADF/COFILIN-1/2 (mentioned collectively as COFILIN) are the only known substrates of LIMKs so far (26). Phosphorylation of COFILIN in Ser3, carried out by phosphorylated LIMK1 (pLIMK1), inhibits its actin severing activity promoting the formation of F-actin. While LIMK1 phosphorylation occurs gradually over the course of the first 60 min of incubation under capacitating conditions, COFILIN displays a transient increase in Ser3 phosphorylation during the first 10 min followed by a rapid return to the basal level (22). These results strongly suggest that the COFILIN phosphorylation status is controlled by other enzymes such as Ser/Thr phosphatases. In somatic cells, Slingshot family of protein phosphatases (SSHs; composed of SSH1, SSH2, and SSH3 in mammals) specifically dephosphorylate and reactivate Ser3-pCOFILIN (27, 28).

We hypothesize that COFILIN is subject to a dual regulation by LIMKs and SSHs in mouse sperm to control its actin-depolymerizing activity. In the present work, we investigated the participation of PAK4 and SSH1 phosphatase in the signaling pathway leading to actin remodeling during mouse sperm capacitation.

## Results

### PAK4 is present in mouse sperm and regulates LIMK1 capacitation-associated phosphorylation

**Figure 1:**
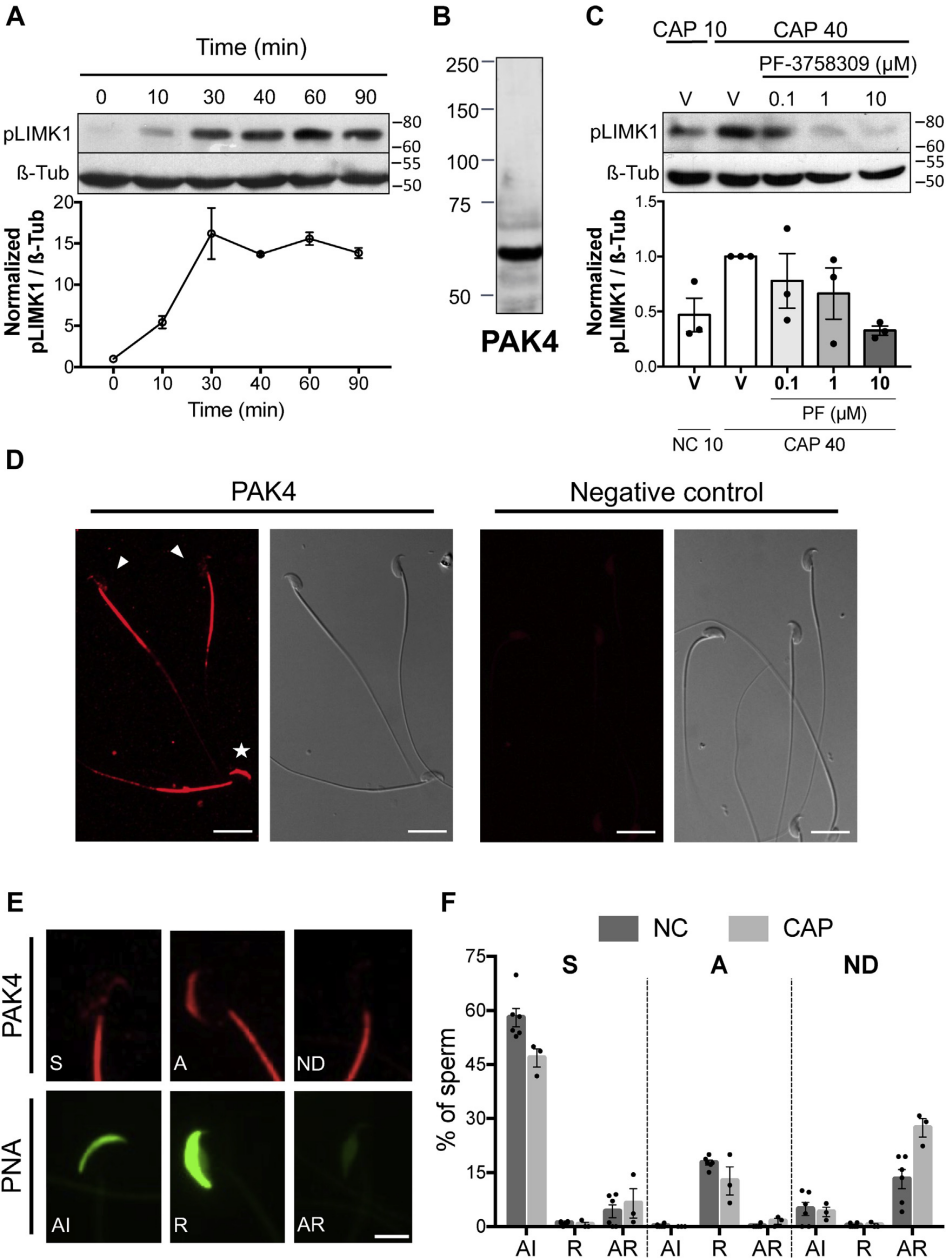
**PAK4 is present in mouse sperm and regulates capacitation associated LIMK1 phosphorylation**. *A*, mouse sperm were incubated for up to 90 min under capacitating conditions. At the indicated time points, proteins were extracted, separated by 8% SDS-PAGE, and immunoblotted with anti-phospho-LIMK1 (Thr508)–specific antibody (pLIMK1). Each lane contains 5 x 10^6^ sperm. As a loading control, anti-β-tubulin was used. Representative images are shown. The image representing the loading control (tubulin) is also used in Figure 5*C* since the membrane was stripped and reused to detect pSSH1. The respective quantitative analysis was performed by measuring the optical density of all bands and relativized to β-tubulin. Results are expressed as the mean ± SEM of at least three independent experiments, where normalization to the 0 min time point was used. *B,* mouse sperm proteins (equivalent to 5 × 10^6^ sperm) were analyzed by 10% SDS-PAGE and immunoblotted using anti-PAK4 specific antibody. *C,* mouse sperm were incubated under capacitating conditions in the absence (V= DMSO) or presence of increasing concentrations of PAK4 inhibitor PF-3758309 (0.1, 1, or 10 μM). The 40 min time point was specifically chosen to study phosphorylation status at the maximum level of pLIMK1. After 10 min (CAP 10) or 40 min (CAP 40) of incubation, proteins were extracted, separated by 10% SDS-PAGE and immunoblotted with anti-pLIMK1 (Thr508) antibody. Each lane contains 5 x 10^6^ sperm. As a loading control, anti-β-tubulin was used. Representative images are shown. The respective quantitative analysis was performed by measuring the optical density of all bands and relativized to β-tubulin. Results are expressed as the mean ± SEM of at least three independent experiments, where normalization to the control condition (V CAP 40) was used. Nonparametric Kruskal–Wallis test was performed in combination with Dunnˊs multiple comparisons test. *D,* representative immunofluorescence images of noncapacitated sperm stained with anti-PAK4 antibody (PAK4). Nonspecific staining was determined by incubating sperm in the absence of primary antibody (negative control). An Alexa Fluor 568-conjugated goat anti-rabbit was used (*red*) and analyzed by confocal microscopy. Right panels correspond to the differential interference contrast (DIC) fields. Scale bar = 15 μm. *E,* representative confocal images of double stained sperm with anti-PAK4 antibody (*red*, *upper panels*) and FITC-conjugated PNA (*green*, lower panels). Three different PAK4 patterns (S = *septum*, A = acrosomal, ND = nondetected) are shown with the respective acrosomal state (AI = acrosome-intact, R = reacting, AR = acrosome-reacted). Scale bar = 5 μm. *F*, the distribution of PNA staining was quantified for each PAK4 pattern in noncapacitated (0 min, NC) and capacitated (60 min, CAP) sperm. Results are expressed as the mean ± SEM of at least three independent experiments. A total of 600 cells were analyzed. Chi-square test indicated that there is a significant association between the distribution of these two variables (PAK4 and PNA patterns; c.i. 95%, *p* < 0.0001). LIMK1, LIM-kinase 1; PAK, p21-activated kinase; pLIMK1, phosphorylated LIMK1; PNA, peanut agglutinin lectin.

In mouse sperm, we previously demonstrated that both RHOA/C and RAC1 small GTPases are necessary for LIMK1 activation. LIMK1 is gradually phosphorylated on Thr508 during capacitation reaching a maximum at 30 min (Figs. 1*A* and S1*A*). In the case of the RHOA/C signaling pathway, we found that ROCK1 is expressed in mouse sperm and participates in the phosphorylation of LIMK1. However, the kinase responsible for RAC1-mediated phosphorylation was not established. The possible participation of PAK1, one of six members of the PAK family of serine/threonine kinases, although expressed in sperm, has been previously excluded (22). We have now explored the involvement of PAK4, another member of the PAK family. By immunoblotting, it was determined that PAK4 is expressed in mouse sperm, and a single band of the expected molecular size (68 KDa) was observed (Fig. 1*B*).

To study whether PAK4 phosphorylates LIMK1, sperm were incubated under capacitating conditions in the presence of increasing concentrations of PAK4-specific inhibitor PF-3758309 (29). Phosphorylation of LIMK1 was significantly reduced in the presence of PAK4 inhibitor (Fig. 1*C*), indicating that this kinase is required for the capacitation-associated LIMK1 activation.

Next, the localization of PAK4 in mouse sperm was evaluated. As shown in Figure 1*D*, immunofluorescence studies revealed the localization of PAK4 mainly along the flagellum midpiece as well as in the head of mouse sperm. Interestingly, PAK4 exhibited three different staining patterns in the sperm head: (1) *septum* (Fig. 1*D*, white arrows; Fig. 1*E*, pattern S), (2) acrosomal (Fig. 1*D*, white asterisk; Fig. 1*E*, pattern A), or (3) absent (Fig. 1*E*, pattern ND). To further understand whether the localization of PAK4 is associated with the acrosomal status, a double staining with peanut agglutinin lectin (PNA) and PAK4 antibody was used in noncapacitating conditions. Sperm displaying the *septum* pattern (60.60 ± 2.99 % of total cells) mainly presented an intact acrosome (91.55 ± 2.73 %; Fig. 1, *E* and *F*, pattern S, NC); while the acrosomal pattern (19.67 ± 1.28 % of total cells) was linked with a more diffuse PNA staining (97.80 ± 1.06 %; Fig. 1, *E* and *F*, pattern A, NC). This last type of staining has been associated with intermediate stages of AR (30). Finally, the absence of PAK4 staining (19.73 ± 1.89 % of total cells) was associated with the lack of acrosome (68.96± 12.38 %; Fig. 1, *E* and *F*, pattern ND, NC). After 60 min of capacitation, the expected increase in spontaneous AR was accompanied by changes in the PAK4 patterns, showing that their association with the acrosomal status was maintained: the 87.82 ± 5.75 % of the *septum* pattern (53,92 ± 5,34 % of total cells) was linked to acrosome intact sperm; the 87.70 ± 8.48 % of the acrosomal pattern (14,19 ± 3.87 % of total cells) was linked to the diffuse PNA staining; and the 86.37 ± 4.13 % of sperm with no PAK4 staining in the head (31,89 ± 3.54 % of total cells) was linked to acrosome reacted sperm (Fig. 1*F*, CAP). Taken together, these results indicate that PAK4 localization in the sperm head is related with different stages of AR development.

### Transient phosphorylation of COFILIN is controlled by RHOA/C pathway and PAK4

**Figure 2:**
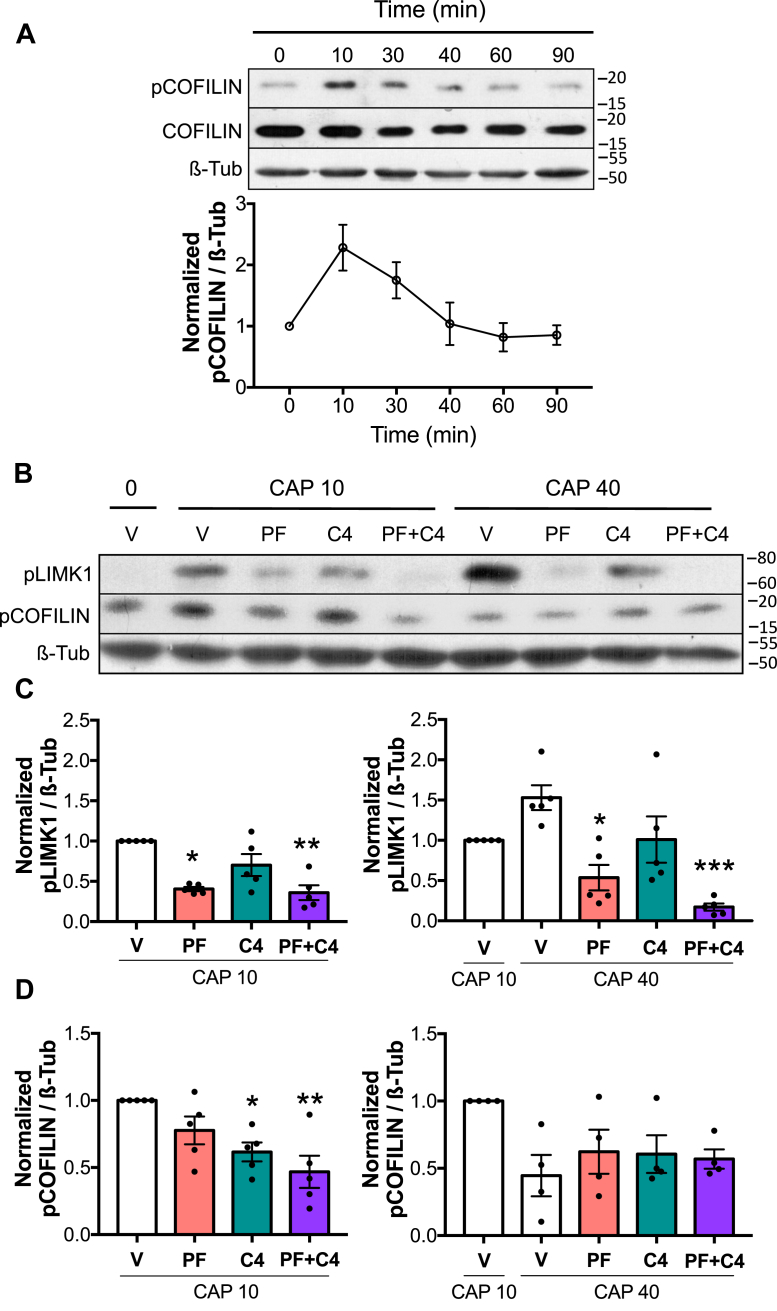
**Transient phosphorylation of COFILIN is controlled by RHOA/C pathway and PAK4.***A,* mouse sperm were incubated for up to 90 min under capacitating conditions. At the indicated times, proteins were extracted, separated by 8 or 12.5% SDS-PAGE, and immunoblotted with anti-phopho-COFILIN (Ser3)– and COFILIN-specific antibodies (pCOFILIN and COFILIN, respectively). Each lane contains 5 × 10^6^ sperm. As a loading control, anti-β-tubulin was used. Representative images are shown. The respective quantitative analysis was performed by measuring the optical density of all bands and relativized to β-tubulin. Results are expressed as the mean ± SEM of at least three independent experiments, where normalization to the 0 min time point was used. *B–D,* mouse sperm were incubated under capacitating conditions in the absence (V= DMSO) or presence of 10 μM PAK4 inhibitor PF-3758309 (PF), 1 μg/ml RHOA/C inhibitor C3 transferase (C4) or both (PF+C4). Protein extracts were performed after 10 min (CAP 10) or 40 min (CAP 40) of incubation, separated by 8 or 12.5% SDS-PAGE, and immunoblotted with anti-pLIMK1 (Thr508)– and anti-pCOFILIN (Ser3)–specific antibodies. Two time points, 10 min and 40 min, were specifically chosen to study phosphorylation status at the maximum level of pCOFILIN and pLIMK1, respectively. As a loading control, anti-β-tubulin was used. *B,* representative blot is shown. Each lane contains 5 × 10^6^ sperm. *C* and *D,* quantitative analysis were performed by measuring the optical density of all bands and relativized to β-tubulin. Results are expressed as the mean ± SEM of at least four independent experiments, where normalization to the control condition (V CAP 10) was used. ∗*p* < 0.05, ∗∗*p* < 0.01, ∗∗∗*p* < 0.001 represent statistical significance between treatments and control at the indicated time, V CAP 10 or V CAP 40. Nonparametric Kruskal–Wallis test was performed in combination with Dunnˊs multiple comparisons test. LIMK1, LIM-kinase 1; PAK, p21-activated kinase; pLIMK1, phosphorylated LIMK1.

The binding of COFILIN to actin is regulated through its phosphorylation on Ser3 by LIMK1, which inhibits its actin-severing activity (31). In mouse sperm incubated under capacitating conditions, while LIMK1 increases its activity gradually over time, the phosphorylated form of COFILIN (pCOFILIN) reaches a transient maximum at 10 min followed by a rapid decrease to the levels comparable to those observed at the beginning of the incubation (Figs. 2*A*; S1, *B* and *C*). These results reveal that COFILIN is subject to a dual modulation in a complex signaling pathway that may involve other regulatory elements.

In previous results, we observed that both RHOA/C and RAC1 small GTPases contribute to LIMK1 and COFILIN phosphorylation (22). Since we identified PAK4 as the kinase acting downstream RAC1, we aimed to elucidate the specific contribution of RHOA/C-ROCK1 and RAC1-PAK4 on COFILIN phosphorylation. For this purpose, sperm were incubated under capacitating conditions in the presence or absence of either PAK4 inhibitor (PF-3758309; 10 μM), RHOA/C inhibitor (esterified C3 transferase, also known as C4; 1 μg/ml), or a combination of both (Fig. 2, *B*–*D*). None of the two inhibitors used in these studies impaired sperm viability (Fig. S2, *A* and *C*, PF and C4, respectively). The phosphorylation status of LIMK1 and COFILIN was evaluated by immunoblotting. Two time points, 10 min and 40 min, were specifically chosen to study the maximum levels of pCOFILIN and pLIMK1, respectively.

As shown in Figure 2, *B* and *C*, phosphorylation of LIMK1 was significantly reduced by PAK4 inhibition, whereas no differences were observed in pCOFILIN maximum at 10 min (Fig. 2*D*; PF). On the other hand, inhibition of RHOA/C resulted in lower levels of pCOFILIN, whereas pLIMK1 was not significantly affected (Fig. 2, *C* and *D*; C4). However, combined treatment resulted in a more pronounced decrease in phosphorylation levels of both proteins (Fig. 2, *C* and *D*; PF+C4). The latter result indicates that PAK4 and RHOA/C are implicated in this phosphorylation cascade.

### PAK4 is an essential player of the Rho GTPases pathway that controls actin polymerization and the AR in mouse sperm capacitation

**Figure 3:**
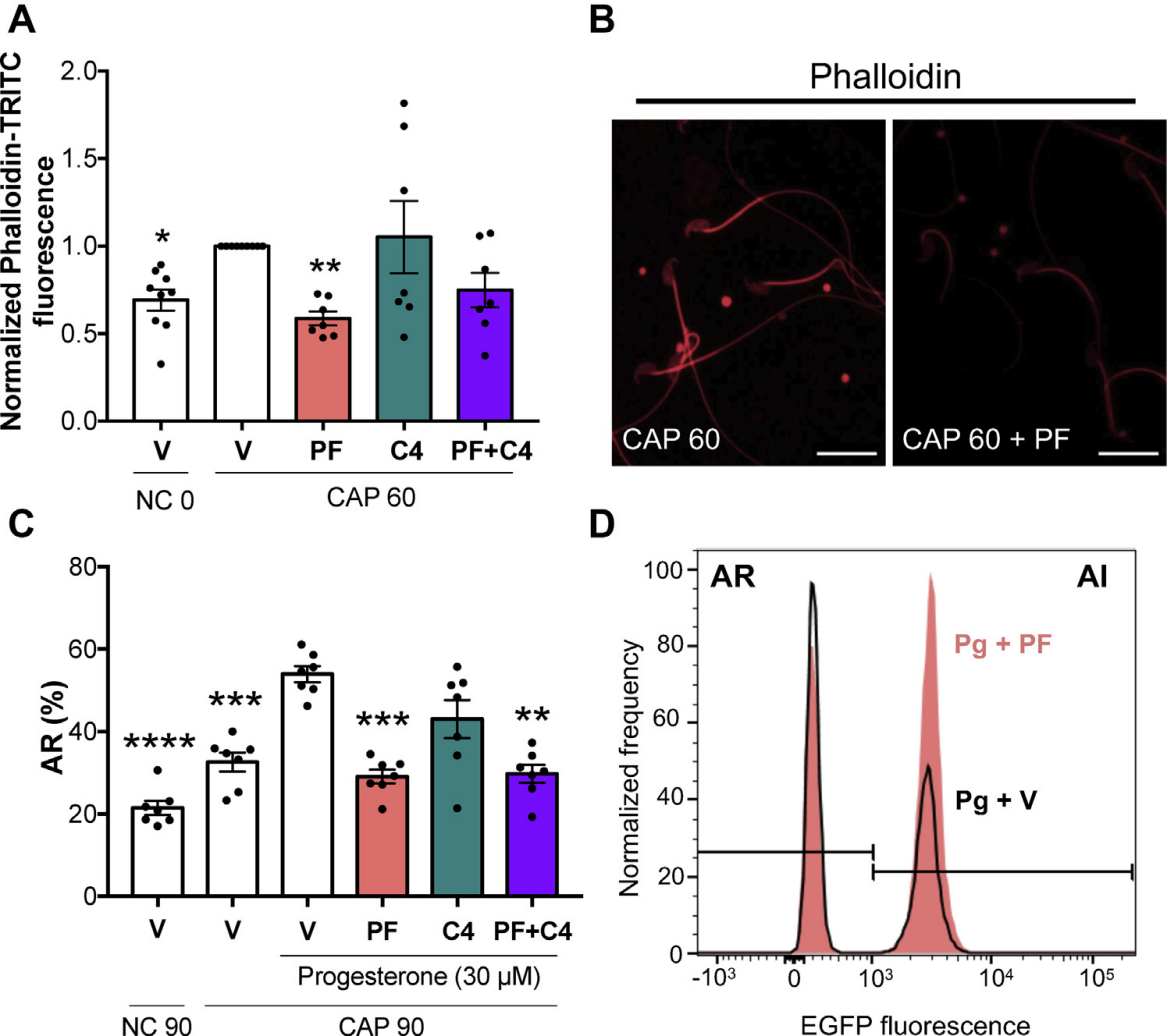
**PAK4 is an essential player of the Rho GTPases pathway that controls actin polymerization and the AR in mouse sperm capacitation**. *A* and *B*, mouse sperm were incubated under noncapacitating (NC) or capacitating (CAP) conditions during 60 min in the absence (V= DMSO) or presence of 10 μM PAK4 inhibitor PF-3758309 (PF), 1 μg/ml RHOA/C inhibitor C4, or both (PF+C4). Fluorescent staining of actin filaments with TRITC-phalloidin was performed and analyzed by confocal microscopy. *A,* quantitative analysis of phalloidin-TRITC fluorescence intensity in the sperm head was performed. Results are expressed as the mean ± SEM of at least eight independent experiments, where normalization to the control condition (V CAP) was used. ∗*p* < 0.05, ∗∗*p* < 0.01 represent statistical significance *versus* control (V CAP). Nonparametric Kruskal–Wallis test was performed in combination with Dunnˊs multiple comparisons test. *B,* representative images of sperm stained by TRITC-phalloidin (*red*) are shown. Left panel: actin polymerization in capacitating conditions. Right panel: actin polymerization in capacitating conditions in the presence of PAK4 inhibitor PF-3758309. Scale bar = 20 μm. *C* and *D,* AR was assessed by flow cytometry analysis of Acr-EGFP sperm. Mouse sperm were incubated for 90 min under noncapacitating (NC) or capacitating (CAP) conditions in the absence (V= DMSO) or presence of 10 μM PAK4 inhibitor PF-3758309 (PF), 1 μg/ml RHOA/C inhibitor C4 or both (PF+C4). At 60 min of incubation, progesterone (30 μM) was added to stimulate AR. Propidium iodide was used to evaluate viability. *C,* percentage of live sperm that undergo AR. Results are expressed as the mean ± SEM of at least seven independent experiments. ∗∗*p* < 0.01, ∗∗∗*p* < 0.001, ∗∗∗∗*p* < 0.0001 represent statistical significance *versus* control (V CAP 90 + Pg). One-way ANOVA was performed in combination with Dunnettˊs multiple comparisons test. *D,* representative experiment using flow cytometry analysis of Acr-EGFP sperm. Histogram analysis depicting normalized frequency of sperm and EGFP fluorescence performed in live sperm populations is shown. AR was induced with progesterone in the absence (black line, Pg + V) or presence of PAK4 inhibitor PF-3758309 (colorful, Pg + PF). AI, acrosome-intact; AR, acrosome-reacted; EGFP, enhanced green fluorescent protein; PAK, p21-activated kinase.

Inhibition of COFILIN by Ser3-phosphorylation, driven by RHOA/C or RAC1, results in a shift of the equilibrium toward polymerization (32, 33). To study the role of PAK4 in actin polymerization, sperm were incubated under capacitating conditions in the presence of RHOA/C or PAK4 inhibitors, fixed, and stained with phalloidin-TRITC to observe F-actin filaments. As shown in Figure 3, *A* and *B*, in the presence of 10 μM PF-3758309 (29), F-actin levels were significantly lower compared to those observed in control condition, evidencing that PAK4 activity is necessary to properly stimulate actin polymerization.

Finally, transgenic mouse sperm expressing enhanced green fluorescent protein (EGFP) in their acrosomes (34) were used to study the impact of PAK4 inhibition on AR. By flow cytometry, we found that inhibition of PAK4 resulted in a significant decrease in the percentage of sperm that underwent AR (Fig. 3, *C* and *D*; PF). These findings are in agreement with previous results by Romarowski *et al.*, where a decreased actin polymerization, caused by a disruption of the Rho GTPase pathway, inhibited AR (22). Interestingly, RHOA/C inhibition (Fig. 3*C*; C4), despite its strong effect on COFILIN phosphorylation (see Fig. 2, *B* and *D*), did not significantly affect AR. Further, combined treatment with PAK4 and RHOA/C inhibitors (Fig 3*C*; PF+C4) inhibited AR at levels comparable to those obtained by 10 μM PF-3758309 alone. This observation, and the fact that PAK4 inhibition had the major effect on actin polymerization in the sperm head, place this kinase as an essential player in AR.

### RAC1 GTPase pathway modulates actin polymerization and AR in mouse sperm

**Figure 4:**
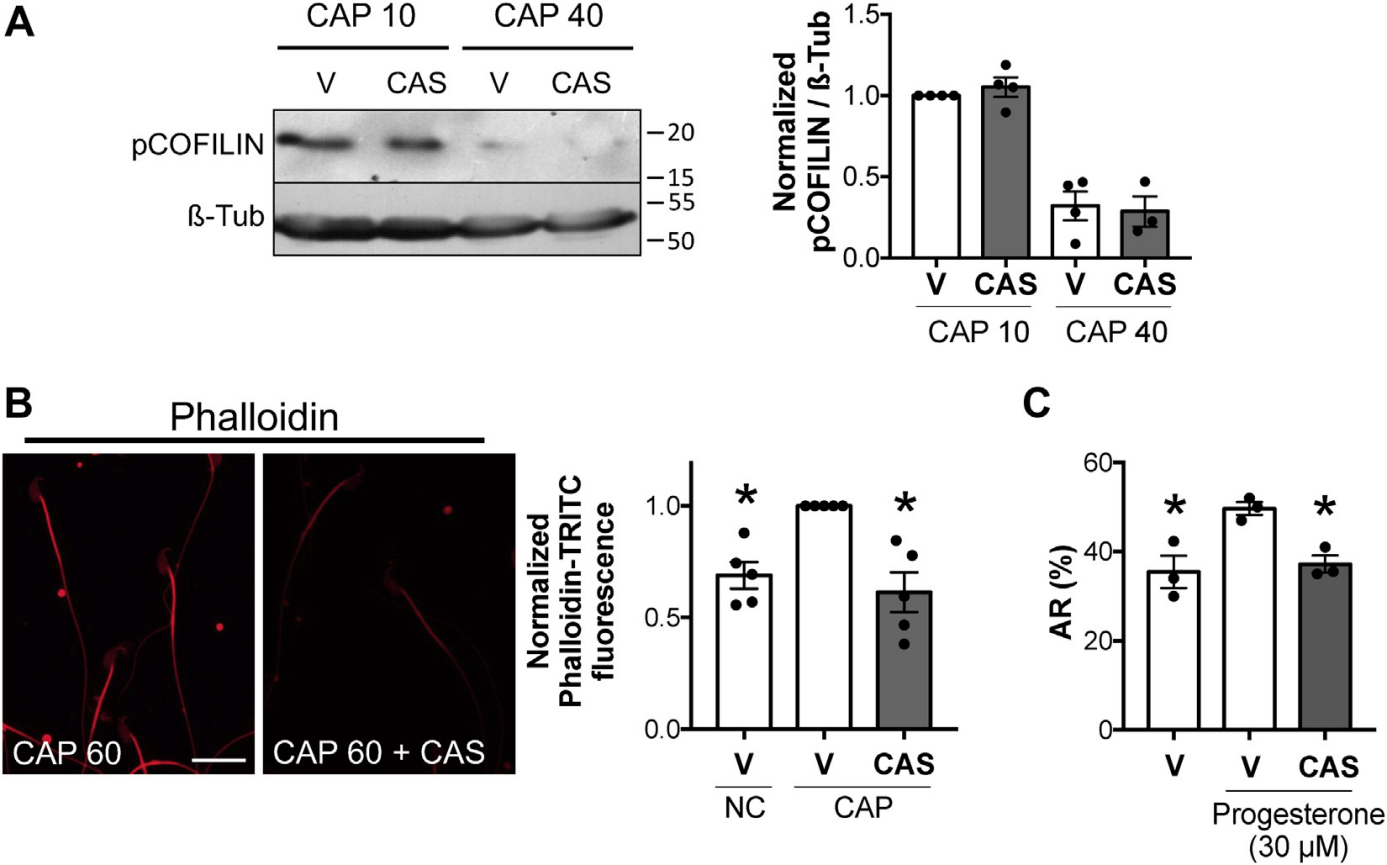
**RAC1 GTPase pathway controls actin polymerization and the AR in mouse sperm capacitation.***A*, mouse sperm were incubated under capacitating conditions in the absence (V= water) or presence of 5 μM RAC1 inhibitor CAS 1177865-17-6 (CAS). Protein extracts were performed after 10 min (CAP 10) or 40 min (CAP 40) of incubation, separated by 12.5% SDS-PAGE, and immunoblotted with anti-pCOFILIN (Ser3)–specific antibody. As a loading control, anti-β-tubulin was used. Each lane contains 5 × 10^6^ sperm. Representative blot is shown. Quantitative analysis was performed by measuring the optical density of all bands and relativized to β-tubulin. Results are expressed as the mean ± SEM of at least three independent experiments, where normalization to the control condition (V CAP 10) was used. Nonparametric Kruskal–Wallis test was performed between treatments and control at the indicated time, V CAP 10 or V CAP 40. *B,* mouse sperm were incubated under capacitating (CAP) conditions during 60 min in the absence (water) or presence of 5 μM RAC1 inhibitor CAS 1177865-17-6 (CAS). Fluorescent staining of actin filaments with TRITC-phalloidin was performed and analyzed by confocal microscopy. Representative images of sperm stained by TRITC-phalloidin (*red*) are shown. Left panel: actin polymerization in capacitating conditions. Right panel: actin polymerization in capacitating conditions in the presence of RAC1 inhibitor CAS 1177865-17-6. Scale bar = 20 μm. Quantitative analysis of phalloidin-TRITC fluorescence intensity in the sperm head was performed. Results are expressed as the mean ± SEM of five independent experiments, where normalization to the control condition (V CAP) was used. ∗*p* < 0.05 represents statistical significance *versus* control (V CAP). Nonparametric Kruskal–Wallis test was performed in combination with Dunnˊs multiple comparisons test. *C,* AR was assessed by Coomassie brilliant blue G-250 staining. Mouse sperm were incubated for 90 min under capacitating (CAP) conditions in the absence (V= water) or presence of 5 μM RAC1 inhibitor CAS 1177865-17-6 (CAS). At 60 min of incubation, progesterone (30 μM) was added to stimulate AR. Results are expressed as the mean ± SEM of three independent experiments. ∗*p* < 0.05 represents statistical significance *versus* control (V + Pg). Arcsine transformation was performed before application of one-way ANOVA with Dunnett's multiple comparisons test. AR, acrosomal exocytosis.

We have previously described that RAC1 contributes to the phosphorylation of LIMK1 (22). Sperm incubation in the presence of RAC1 inhibitor (CAS 1177865–17–6; 5 μM) did not affect pCOFILIN levels (Fig. 4*A*). However, it significantly reduced actin polymerization (Fig. 4*B*) and abrogated AR (Fig. 4*C*), without impairing viability (Fig. S2*B*, CAS). These results are similar to those obtained with PAK4 inhibitor PF-3758309, suggesting that PAK4 may be the downstream effector of RAC1. This possibility needs to be explored in future investigations.

### SSH1 phosphatase is present in mouse sperm and is phosphorylated in Ser978 during capacitation

**Figure 5:**
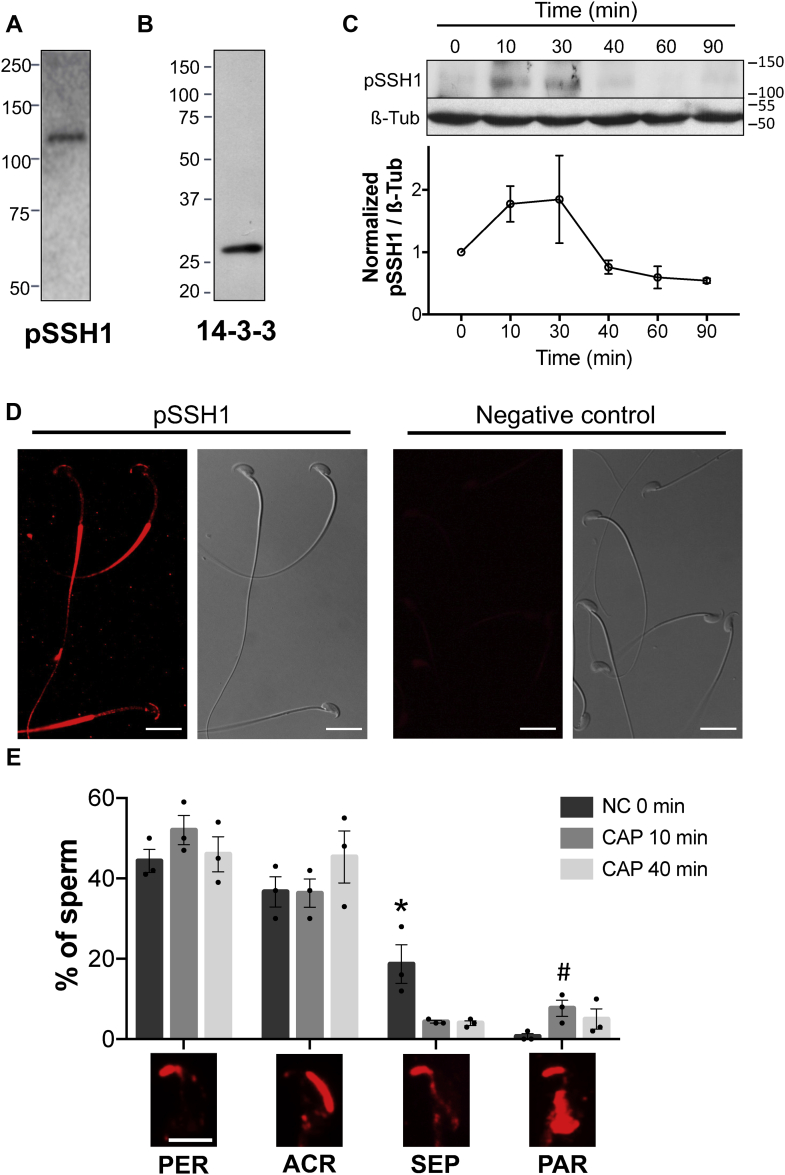
**SSH1 phosphatase is present in mouse sperm and is phosphorylated in Ser978 during capacitation**. *A* and *B*, mouse sperm proteins (equivalent to 5 × 10^6^ sperm) were analyzed by 8 or 12.5% SDS-PAGE and immunoblotted using (*A*) anti-phospho-SSH1 (Ser978; pSSH1) or *(B)* anti-pan-14-3-3 antibodies. *C,* mouse sperm were incubated for up to 90 min under capacitating conditions. At the indicated times, proteins were extracted, separated by 8% SDS-PAGE and immunoblotted with anti-pSSH1 (Ser978)–specific antibody. Each lane contains 5 × 10^6^ sperm. As a loading control, anti-β-tubulin was used. Representative images are shown. The image representing the loading control (tubulin) is also used in Figure 1*A* since the membrane was stripped and reused to detect pLIMK1. The respective quantitative analysis was performed by measuring the optical density of all bands and relativized to β-tubulin. Results are expressed as the mean ± SEM of at least three independent experiments, where normalization to the 0 min time point was used. *D,* representative immunofluorescence images of sperm stained with anti-pSSH1 antibody. Nonspecific staining was determined by incubating sperm in the absence of primary antibody (negative control). An Alexa Fluor 568-conjugated goat anti-rabbit was used (*red*) and analyzed by confocal microscopy. Right panels correspond to the differential interference contrast fields. Scale bar = 15 μm. *E,* the percentage of sperm exhibiting a pSSH1 pattern (PER= *perforatorium*, ACR= acrosomal, SEP= *septum,* and PAR= postacrosomal region) considered individually was quantified for each time point: 0, 10, and 40 min of capacitation. Results are expressed as the mean ± SEM of three independent experiments. A total of 500 cells were analyzed. Scale bar = 5 μm. For each pattern one-way ANOVA was performed in combination with Tukeyˊs multiple comparisons test. ∗*p* < 0.05 represents statistical significance. A Student's *t* test was applied to compare between conditions 0 and 10 min of the PAR pattern. #*p* > 0.05 represents statistical significance. pLIMK1, phosphorylated LIMK1; SSH, Slingshot family of protein phosphatase.

As previously shown, phosphorylation of LIMK1 on Thr508 increases gradually during capacitation (Fig. 1*A*), while phosphorylation of COFILIN on Ser3 reaches a maximum at 10 min and then returns to baseline levels (see Fig. 2*A*). These results suggest the participation of an alternative player implicated in the regulation of COFILIN phosphorylation. In somatic cells, dephosphorylation and consequent activation of pCOFILIN is mediated by SSH1 phosphatase (27, 35, 36). Thus, the presence of SSH1 in mouse sperm was analyzed by immunoblotting using a specific antibody against the phosphorylated form of this protein (pSSH1). As shown in Figure 5*A*, a single band of the expected size for pSSH1 (116 kDa) was observed. Furthermore, we found that 14-3-3, a scaffold protein implicated in SSH1 regulation (36), is also expressed in mouse sperm (Fig. 5*B*).

Phosphorylation of SSH1 on Ser978 is known to inhibit its phosphatase activity (35). In order to study the phosphorylation status of SSH1 during capacitation, sperm were incubated under capacitating conditions, and the levels of pSSH1 at different times of incubation were analyzed. As shown in Figure 5*C*, SSH1 is phosphorylated within the first 10 min of incubation, remains sustained until 30 min, and then decays to the levels observed at the beginning of the incubation.

Immunofluorescence studies revealed the localization of pSSH1 mainly along the principal piece of the flagellum as well as in different regions of the sperm head (Fig. 5, *D* and *E*): *perforatorium* (Fig. 5*E*, PER), acrosomal (Fig. 5*E*, ACR), *septum* (Fig. 5*E*, SEP), and postacrosomal region (Fig. 5*E*, PAR). These four patterns were found alone or combined, being the most representative the *perforatorium* together with the acrosomal, as shown in Figure 5*D*. To study the localization of pSSH1 during capacitation, the time points 0, 10, and 40 min were chosen. Two of the patterns, the SEP and the PAR, exhibited a specific dynamic within the first 10 min of capacitation (Fig. 5*E*, SEP and PAR patterns). The *septum* pattern significantly decreased with 10 min of capacitation, whereas the postacrosomal showed a tendency to increase.

### pCOFILIN dephosphorylation by SSH1 phosphatase is not essential for actin polymerization or AR

**Figure 6:**
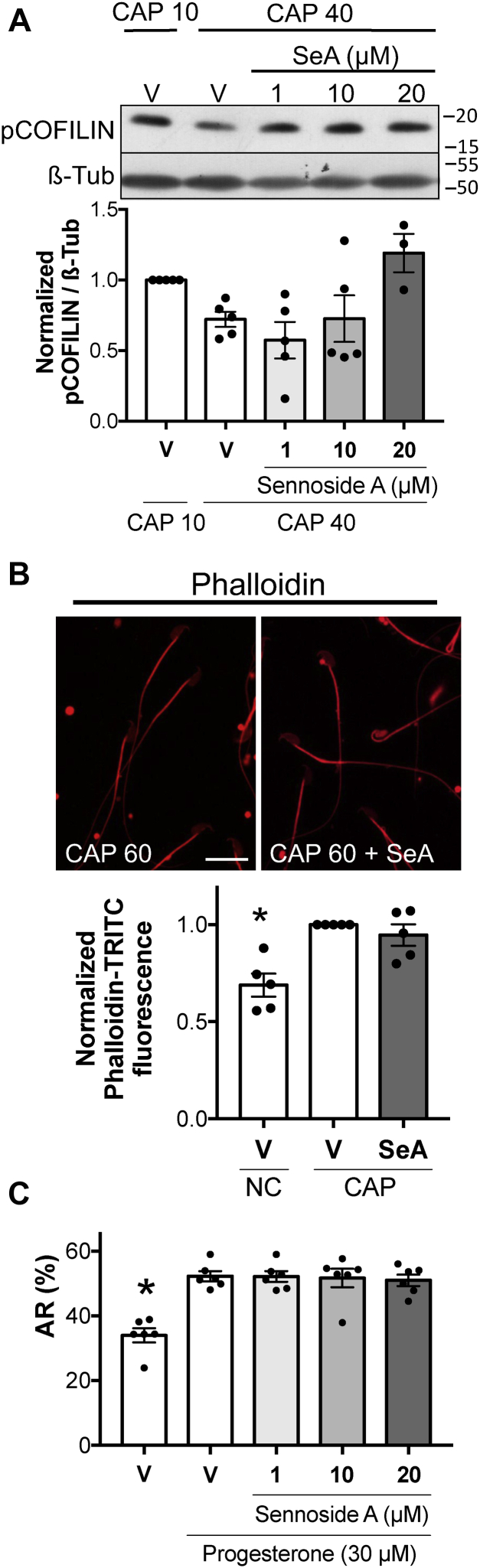
**pCOFILIN dephosphorylation by SSH1 phosphatase is not essential for actin polymerization or AR**. *A,* mouse sperm were incubated under capacitating conditions in the absence (V= DMSO) or presence of increasing concentrations of SSH1 inhibitor sennoside A (SeA; 1, 10 or 20 μM). At the indicated times, proteins were extracted, separated by 12.5% SDS-PAGE, and immunoblotted with anti-pCOFILIN (Ser3) antibody. Each lane contains 5 x 10^6^ sperm. As a loading control, anti-β-tubulin was used. Representative images are shown. The respective quantitative analysis was performed by measuring the optical density of all bands and relativized to β-tubulin. Results are expressed as the mean ± SEM of at least three independent experiments, where normalization to the control condition (V CAP 10) was used. Nonparametric Kruskal–Wallis test was performed in combination with Dunnˊs multiple comparisons test. *B,* mouse sperm were incubated under capacitating (CAP) conditions during 60 min in the absence (DMSO) or presence of SSH1 inhibitor SeA (20 μM). Fluorescent staining of actin filaments with TRITC-phalloidin was performed and analyzed by confocal microscopy. Representative images of sperm stained by TRITC-phalloidin (*red*) are shown. Left panel: actin polymerization in capacitating conditions. Right panel: actin polymerization in capacitating conditions in the presence of SSH1 inhibitor SeA (20 μM). Scale bar = 20 μm. Quantitative analysis of phalloidin-TRITC fluorescence intensity in the sperm head was performed. Results are expressed as the mean ± SEM of five independent experiments, where normalization to the control condition (V CAP) was used. ∗*p* < 0.05 represents statistical significance *versus* control (V CAP). Nonparametric Kruskal–Wallis test was performed in combination with Dunn ˊs multiple comparisons test. *C,* AR was assessed by flow cytometry analysis of Acr-EGFP sperm. Mouse sperm were incubated for 90 min under capacitating conditions in the absence (V= DMSO) or presence of SSH1 inhibitor SeA (1, 10 or 20 μM). At 60 min of incubation, progesterone (30 μM) was added to stimulate AR. Propidium iodide was used to evaluate viability. Percentage of live sperm that undergo AR is shown. Results are expressed as the mean ± SEM of at least six independent experiments. ∗*p* < 0.05 represent statistical significance *versus* control (V + Pg). One-way ANOVA was performed in combination with Dunnett ˊs multiple comparisons test. AR, acrosomal exocytosis; EGFP, enhanced green fluorescent protein; SSH, Slingshot family of protein phosphatase.

Given that the decrease in pSSH1 is coincident with the diminish in pCOFILIN levels, these results suggest that SSH1 could be the phosphatase responsible for pCOFILIN dephosphorylation. To investigate this possibility, sperm were incubated under capacitating conditions in the absence or presence of a SSH1-specific inhibitor sennoside A (37). As shown in Figure 6*A*, the highest concentration of this inhibitor resulted in persistent COFILIN phosphorylation at 40 min of incubation. In this condition, sperm viability was not compromised (Fig. S2*A*, SeA). Surprisingly, sennoside A did not affect actin polymerization (Fig. 6*B*) or AR (Fig. 6*C*). Taken together, these results suggest that capacitation-associated actin polymerization and the AR depend on early phosphorylation of COFILIN but not on its subsequent dephosphorylation.

### Okadaic acid–sensitive phosphatases mediate SSH1 activation

**Figure 7:**
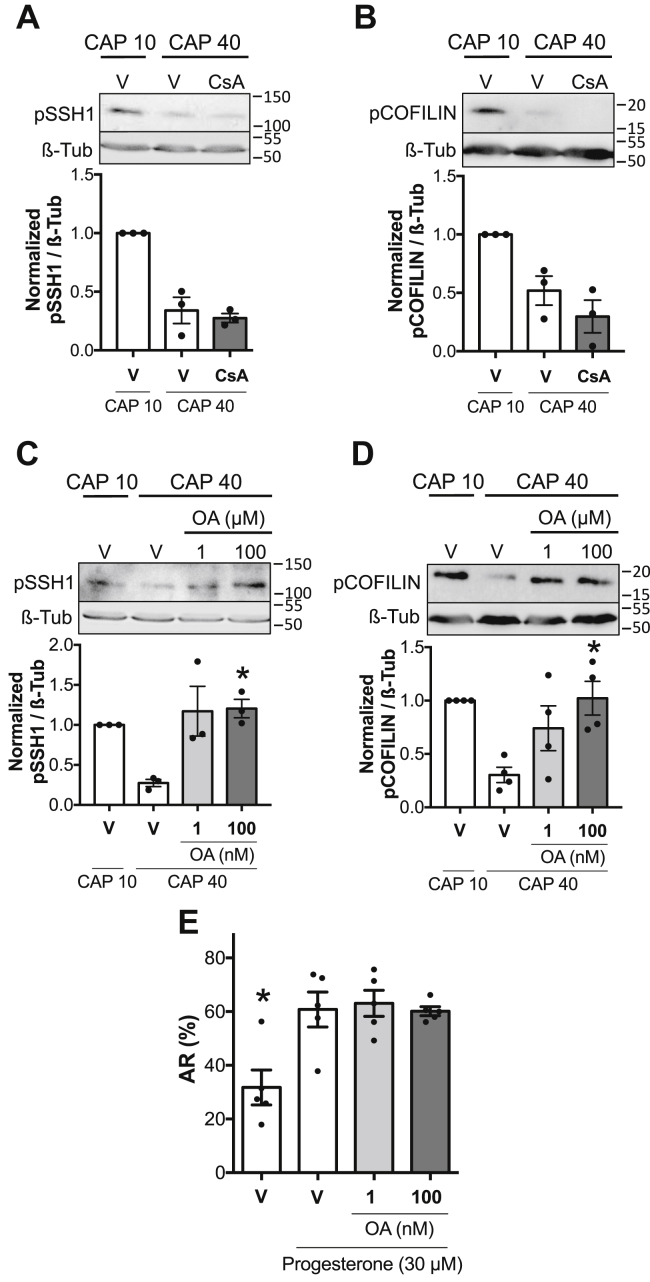
**Okadaic acid-sensitive phosphatases mediate SSH1 activation**. *A–D*, mouse sperm were incubated under capacitating conditions in the absence (V= DMSO) or presence of 5 μM cyclosporin A (CsA) or one or 100 nM okadaic acid (OA). At the indicated times, proteins were extracted, separated by 8 or 12.5% SDS-PAGE and immunoblotted with anti-pSSH1 (Ser978) or anti-pCOFILIN (Ser3) antibodies. Each lane contains 5 x 10^6^ sperm. As a loading control, anti-β-tubulin was used. Representative images are shown. The respective quantitative analysis was performed by measuring the optical density of all bands and relativized to β-tubulin. Results are expressed as the mean ± SEM of at least three independent experiments, where normalization to the control condition (V CAP 10) was used. ∗*p* < 0.05 represent statistical significance *versus* control (V CAP 40). Nonparametric Kruskal–Wallis test was performed in combination with Dunnˊs multiple comparisons test. *E*, AR was assessed by flow cytometry analysis of Acr-EGFP sperm. Mouse sperm were incubated for 90 min under capacitating (CAP) conditions in the absence (V= DMSO) or presence of OA (1 or 100 nM). At 60 min of incubation, progesterone (30 μM) was added to stimulate AR. Propidium iodide was used to evaluate viability. Percentage of live sperm that undergo AR is shown. Results are expressed as the mean ± SEM of five independent experiments. ∗*p* < 0.05 represent statistical significance *versus* control (V + Pg). One-way ANOVA was performed in combination with Dunnettˊs multiple comparisons test. AR, acrosomal exocytosis; EGFP, enhanced green fluorescent protein.

Several studies in somatic cells have shown that pCOFILIN dephosphorylation is a Ca^2+^-induced event mediated by PP2B (also known as calcineurin)-dependent activation of SSH1 (38, 39). To investigate if the phosphatase PP2B controls SSH1 activity by regulating its phosphorylation in Ser978, sperm were incubated under capacitating conditions in the absence or presence of PP2B-specific inhibitor cyclosporin A (CsA; 5 μM). As shown in Figure 7*A*, CsA did not prevent the phosphorylation decrease observed in SSH1 at 40 min, indicating that activation of SSH1 is independent of PP2B. As expected, pCOFILIN levels were also unaffected in the presence of CsA (Fig. 7*B*).

Okadaic acid (OA)–sensitive Ser/Thr phosphatases, PP1 and PP2A, were reported to participate in the regulation of crucial events that occur during sperm capacitation (40, 41). Since it was found that PP2B does not participate in SSH1 regulation, we proceeded to study whether these phosphatases are involved. Sperm were incubated in capacitating conditions in the absence or presence of 1 nM OA, reported to inhibit PP2A only, or 100 nM OA, concentration in which both PP1 and PP2A are blocked (42, 43, 44). As shown in Figure 7*C*, dephosphorylation of pSSH1 was strongly inhibited in the presence of both OA concentrations, indicating that PP2A phosphatase is involved in the regulation of SSH1. The persistent phosphorylation of pSSH1 was reflected in pCOFILIN, whose maximum phosphorylation was maintained at 40 min (Fig. 7*D*). None of the inhibitors used in these studies impaired the viability of the cells during the incubation (Fig. S2, *A* and *C*, OA and CsA, respectively).

Finally, flow cytometry analysis was performed to study AR in these conditions. Consistent with the effect observed with sennoside A, the presence of OA did not affect the occurrence of AR (Fig. 7*E*).

### SSH1 phosphorylation during early capacitation is controlled by RHOA/C and RAC1 pathways.

**Figure 8:**
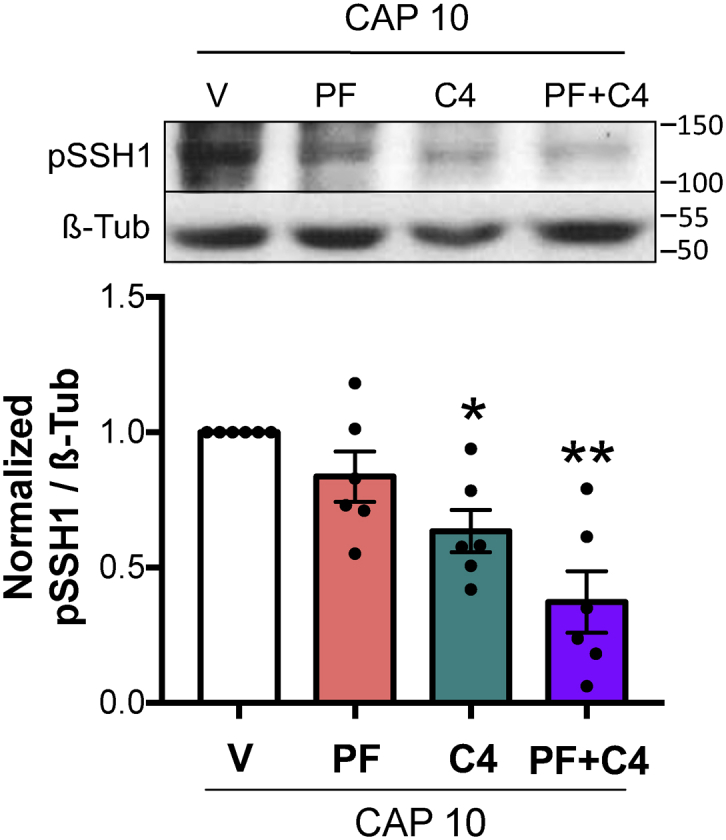
**SSH1 phosphorylation during early capacitation is controlled by RHOA/C and RAC1 pathways**. Mouse sperm were incubated under capacitating conditions in the absence (V= DMSO) or presence of 10 μM PAK4 inhibitor PF-3758309 (PF), 1 μg/ml RHOA/C inhibitor C3 transferase (C4), or both (PF+C4). Protein extracts were performed after 10 min (CAP 10) of incubation, separated by 8% SDS-PAGE, and immunoblotted with anti-pSSH1 (Ser978) specific antibody. As a loading control, anti-β-tubulin T4026 was used. Representative blot of at least four independent experiments is shown. Quantitative analysis was performed by measuring the optical density of all bands and relativized to β-tubulin. Results are expressed as the mean ± SEM of at least four independent experiments, where normalization to the control condition (V CAP 10) was used. ∗*p* < 0.05, ∗∗*p* < 0.01 represent statistical significance between treatments and control (V CAP 10). Nonparametric Kruskal–Wallis test was performed in combination with Dunnˊs multiple comparisons test. PAK, p21-activated kinase; SSH, Slingshot family of protein phosphatase.

In another layer of regulation, previous reports postulate that SSH1 phosphorylation is under control of the same kinases that lead LIMK1 and COFILIN phosphorylation. In particular, it is well-known that in somatic cells PAK4 can accomplish this task after its activation by RAC1 GTPase pathway (35, 36). In order to elucidate if this regulation is present in mouse sperm, PAK4 inhibitor (PF-3758309; 10 μM), RHOA/C inhibitor (C4; 1 μg/ml), or a combination of both were used following the same experimental design described above. Inhibition of RHOA/C but not PAK4 resulted in lower levels of pSSH1, and the combined treatment decreased phosphorylation levels more significantly than 1 μg/ml C4 alone, indicating the existence of compensatory events (Fig. 8).

In the same direction of our previous results, the similarities between SSH1 and COFILIN phosphorylation responses to these treatments place the phosphatase as a major regulator of COFILIN activity, as in conditions in which SSH1 phosphorylation was affected (Fig. 8), pCOFILIN was significantly decreased independently of LIMK1 phosphorylation state (Fig. 2, *C* and *D*).

## Discussion

One of the endpoints of sperm capacitation is the acquisition of the ability to undergo AR prior to the interaction with the egg. To trigger this exocytotic process, a precise interwind of molecular pathways needs to occur in the sperm head to promote the release of the acrosomal content. One of the key players in facilitating this priming event is the reorganization of the actin cytoskeleton. In this initial step, it was demonstrated in several mammalian species that there is a clear increase in actin polymerization in the sperm head (16). In this work, we demonstrate that in mouse sperm, the small GTPase RAC1 and the kinase PAK4 are essential for actin polymerization and AR during the early molecular events of capacitation.

The ADF/COFILIN family of actin-binding proteins in eukaryotes has long been known to play a key role in actin-filament dynamics. Previous reports in human sperm have shown that COFILIN is important for actin dynamics (45) and that its expression correlates with sperm quality (46). Phosphorylation of COFILIN on Ser3 by activated LIMK1 is the best characterized way of regulation. Thus, LIMK1 modulation is a key factor in the COFILIN phosphoregulatory switch mechanism. It is widely accepted that phosphorylated COFILIN is inactive and no longer binds or severs F-actin resulting in increased F-actin formation. However, a recent report has demonstrated by *in vitro* studies that pCOFILIN binds and weakly severs actin filaments, suggesting that it is not fully inactive (47).

We have previously shown that in mouse sperm, LIMK1 is gradually phosphorylated downstream RHOA/C and RAC1 (22). In the RHOA/C pathway, the classic kinase ROCK1 is responsible for LIMK1 phosphorylation. However, the kinase acting downstream RAC1 activation remained unsolved. Commonly, LIMK1 is a target of RAC1 through PAK1, PAK2, and PAK4. In our previous work, we already ruled out the participation of PAK1 in this pathway (22). Here, we showed that PAK4 is the kinase in charge of LIMK1 phosphorylation. From a functional point of view, PAK4 inhibition strongly reduced pLIMK1 levels. However, because both RHOA/C and RAC1 participate in LIMK1 phosphorylation, only when both pathways are inhibited, COFILIN phosphorylation is completely abolished. This agrees with previous results from our laboratory where it was observed that it is necessary to include pharmacological inhibitors for RHOA/C and RAC1 GTPases to impede LIMK1 and COFILIN phosphorylation (22). Furthermore, we report that PAK4 is strongly expressed in the sperm flagellum and head. Interestingly, two main PAK4 patterns are observed in the head. When a marker of acrosomal integrity was used, we determined that those sperm with intact acrosome display either a post acrosomal staining or over the anterior arc region. However, once AR has occurred, the signal in the head is completely lost. It is known that AR involves dynamic modifications of many structures (*i.e.,* actin structures) and proteins present in the sperm head (15, 48). Our findings suggest that the observed changes in the localization of PAK4 may be associated with the development of AR as they accompanied when spontaneous AR increased in capacitating conditions, but further experiments are needed to test this hypothesis. Inhibition of PAK4 alone alters the capacitation-induced actin polymerization as well as the progesterone-stimulated AR. On the other hand, in agreement with those results reported by Ramírez-Ramírez *et al*., 2020 in guinea pig (25), pharmacological inhibition of RAC1 also decreased the levels of actin polymerization and the percentage of cells that underwent AR in mouse. As the same phenotype was observed by blocking either PAK4 or RAC1, PAK4 might also be an effector of RAC1 in mouse sperm. Altogether, these results suggest that the signaling pathway leading to PAK4 activation is critical for the dynamic reorganization of the actin cytoskeleton necessary for exocytosis.

The activating LIMK1 phosphorylation increases during capacitation, reaching a maximum level at around 30 min of incubation. However, the levels of phosphorylated COFILIN increased transiently during capacitation, reaching a maximum at 10 min of incubation and returning to basal levels after 30 min. This observation suggests that COFILIN is subject to other regulatory mechanism. In somatic cells, COFILIN is inactivated by LIMK1-catalyzed phosphorylation and reactivated by SSH1-mediated dephosphorylation (36). Our results suggest that this mechanism is conserved in mouse sperm. We found that SSH1 is expressed in mouse sperm and also displays a transient phosphorylation during the first 30 min of incubation under capacitating conditions. The time course analysis of both pCOFILIN and pSSH1 is compatible with the regulatory role of SSH1 on COFILIN. The maximum levels of the inactive form of SSH1 (phosphorylated in Ser978) are coincident with the transient increase in COFILIN phosphorylation. Then, pSSH1 is dephosphorylated, and its phosphatase activity increases, resulting in a decrease in pCOFILIN. These results are consistent with our pharmacological experiments. When sperm were exposed to the SSH1 inhibitor sennoside A, the transient increase in pCOFILIN is abolished, and pCOFILIN remains phosphorylated in later times.

In other biological systems, it is observed that PP2B (calcineurin) is responsible for pSSH1 dephosphorylation (38, 39). However, inhibition of PP2B by cyclosporin A did not affect either pSSH1 or pCOFILIN levels. To investigate if phosphatases sensitive to OA are involved in pSSH1 dephosphorylation, phosphorylation status of SSH1 and COFILIN was assessed in the presence of different concentrations of OA. The fact that the very low concentration of OA (IC50: 1 nM) was capable of inhibiting pCOFILIN dephosphorylation points toward the participation of the phosphatase PP2A in this pathway, which is about 100-fold more sensitive to OA than PP1 (42, 43, 44). It is noteworthy that these phosphatases are the two main Ser/Thr phosphatases present in the sperm (49). To the best of our knowledge, the regulatory mechanism of SSH1 through PP2A is unique of mouse sperm and remains to be investigated in other mammalian species.

Dephosphorylation of pCOFILIN occurring after 10 min under capacitating conditions is not essential for actin polymerization or AR. Even when pCOFILIN persists phosphorylated by either direct inhibition of SSH1 by sennoside A or by using OA, sperm undergo AR normally. We hypothesize that actin polymerization is controlled by early and late molecular events. While COFILIN is essential during the early steps of capacitation, other players may control this process later. Remarkably, these two stages seem to be linked, since the early phosphorylation of COFILIN is a requirement for the increase of actin polymerization at later incubation times under capacitating conditions. One possibility is that pCOFILIN itself stimulates phospholipase D1 (PLD1) activity as demonstrated in other cellular systems (50). Furthermore, the participation of PLD1 in actin polymerization in mouse and human sperm has been reported (51). Future studies are needed to elucidate how the LIMK1/COFILIN signaling pathway interacts with PLD1.

While in some cell types, a given stimulation induces changes in net pCOFILIN levels, in other cells, a robust increase in pCOFILIN turnover was observed with no significant change in the total pCOFILIN pool (52). The latter observations suggested that the activity of COFILIN kinases and phosphatases targeting COFILIN are being regulated by common upstream stimulatory signals. Our findings demonstrate that LIMK1 activation and SSH1 inhibition are triggered simultaneously, downstream of RHOA/C and RAC1 effectors. This observation probably explains the robust COFILIN phosphorylation. Further, previous evidence showed that these players can associate forming a complex, together with the scaffold protein 14-3-3 and actin filaments, favoring its coordinated regulation (53, 54).

The presence of CDC42, another member of the Rho-family of small GTPase proteins (together with RAC, and RHO), and some of their downstream effectors has been described in mammalian sperm (55, 56, 57). It has been reported to be essential for actin polymerization and the development of AR in guinea pig and mouse sperm (58). In addition, CDC42 is involved in the modulation of CatSper activity and other calcium-dependent downstream events in mouse sperm (59).

**Figure 9:**
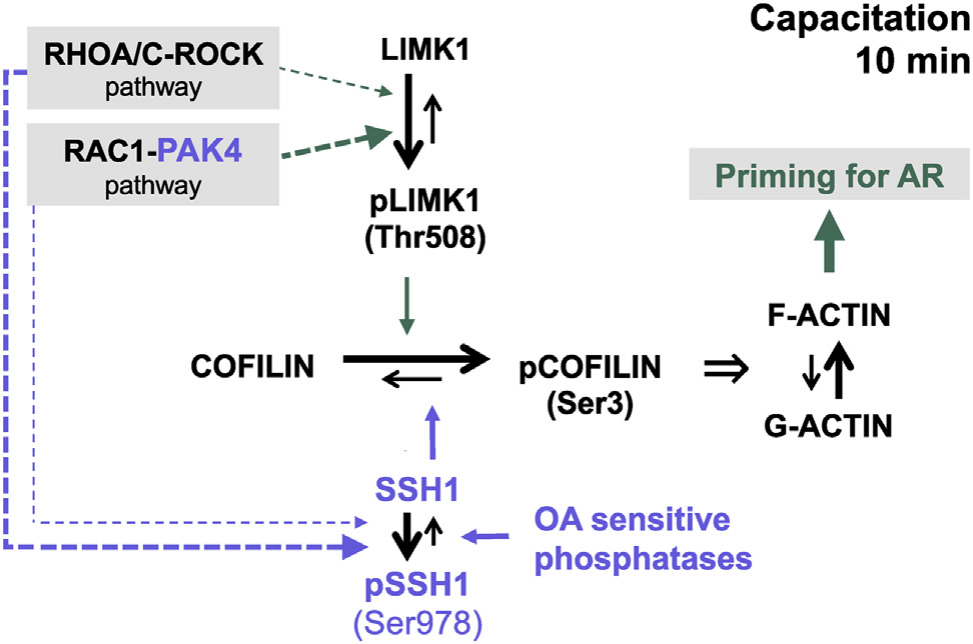
**Working model**. Stimulation of RHOA/C-ROCK1 and RAC1-PAK4 pathways during early steps of capacitation induces LIMK1 activation and SSH1 phosphatase inhibition. The consequent phosphorylation of COFILIN in Ser3 inhibits its actin severing activity allowing the first actin polymerization event. This signaling cascade is an essential step for preparing the sperm to undergo AR. The thickness of the dashed arrows indicates the magnitude of the contribution of that pathway. AR, acrosomal exocytosis; LIMK1, LIM-kinase 1; PAK, p21-activated kinase; pLIMK1, phosphorylated LIMK1; ROCK, Rho-associated activated kinase; SSH, Slingshot family of protein phosphatase.

Altogether, we propose the following working model presented in Figure 9. Although both, the activity of RHOA/C and RAC1 GTPases are essential for LIMK1 activation and COFILIN phosphorylation, the most significant contribution to the downstream effects such as actin polymerization and the AR is provided by RAC1 and PAK4. On the other hand, the turn on of these GTPases also promote the inactivation of the COFILIN phosphatase SSH1. Later on, SSH1 is triggered by OA phosphatases that result in pCOFILIN dephosphorylation. The events leading to COFILIN phosphorylation at the early stages of capacitation are essential for actin polymerization and the ability to undergo AR.

## Experimental procedures

### Reagents

Bovine serum albumin (BSA) A7906, progesterone, Coomassie brilliant blue-G, sennoside A, TRITC-labeled phalloidin, fluorescein isothiocyanate (FITC)-labeled PNA, phosphatase inhibitor cocktail P5726, and protease inhibitor cocktail P8340 were purchased from Sigma-Aldrich. Eosin Y was purchased from Biopack. Membrane-permeable Exo enzyme C3 Transferase (C4) was obtained from Cytoskeleton. CAS 1177865-17-6 was obtained from Merck. Cyclosporin A and OA were obtained from Cayman Chemical. Horseradish peroxidase (HRP)-conjugated anti-rabbit IgG and HRP anti-mouse IgG from Vector Laboratories. PF-3758309 from Medkoo Biosciences. Antibody anti-rabbit IgG-Alexa Fluor 568 from Invitrogen, Thermo Fisher Scientific; while anti-14-3-3 (pan 14-3-3 b8) from Santa Cruz Biotechnology. Anti-COFILIN, anti-phospho-SSH1 (pSSH1; Ser978), anti-phospho-LIMK1/2 (pLIMK1/2; Thr508), and anti-phospho-COFILIN (pCOFILIN; Ser3) antibodies were purchased from Cell Signaling. Anti-PAK4 from Proteintech. Anti-β-tubulin E7 was obtained from Developmental Studies Hybridoma Bank University of Iowa. PF-3758309, cyclosporine A, OA, and sennoside A were dissolved in DMSO; C4, CAS 1177865-17-6, and Eosin Y were dissolved in hexa-distilled water, and phalloidin-TRITC and PNA-FITC were dissolved in PBS.

### Animals

Hybrid F1 (BALB/c x C57BL/6) (10–12-weeks-old) male mice as well as mature transgenic [B6D2F1-Tg (CAG/mt-DsRed2, Acr-EGFP) RBGS002Osb] (34) were used. Mice were housed in groups of four in a temperature-controlled room (23 °C) with lights on at 07:00 AM and off at 7:00 PM and had free access to tap water and laboratory chow. All experimental procedures were carried according to guidelines of the institutional animal care and were reviewed and approved by the Ethical Committees of the *Instituto de Biología y Medicina Experimental, Buenos Aires* (#CE/003–1/2011)*.* Experiments were performed in strict accordance with the Guide for Care and Use of Laboratory Animals approved by the National Institutes of Health.

### Sperm medium and capacitation

The noncapacitating medium used in this study was a modified Toyoda–Yokoyama–Hosi (modified TYH) containing 119.3 mM NaCl, 4.7 mM KCl, 1.71 mM CaCl_2_.2H_2_O, 1.2 mM KH_2_PO_4_, 1.2 mM MgSO_4_.7H_2_O, 0.51 mM sodium pyruvate, 5.56 mM glucose, 20 mM Hepes, and 10 μg/ml gentamicin (NC medium). For capacitating conditions, 15 mM NaHCO_3_ and 5 mg/ml BSA were added (CAP medium). In all cases, pH was adjusted to 7.4 with NaOH.

Animals were euthanized and cauda epididymal mouse sperm were collected and placed in 1 ml of noncapacitating–modified TYH medium (NC: without BSA and NaHCO_3_). After 15 min of incubation at 37 °C (swim-out), epididymis were removed, and sperm were resuspended to a final maximum concentration of 1 × 10^7^ cells/ml on the appropriate medium, depending on the experiment performed. A 10 min preincubation in NC medium containing SSH1 inhibitor (sennoside A), PAK4 inhibitor (PF-3758309), RHOA/C GTPase inhibitor (C4), Rac1 GTPase inhibitor (CAS 1177865–17–6), PP2B inhibitor (cyclosporin A), or PP2A/PP1 inhibitor (OA) was conducted when required. An equal volume of NC or two-fold concentrated capacitating medium (CAP 2X: 30 mM NaHCO_3_ and 10 mg/ml BSA) containing the appropriate inhibitors was added. Finally, sperm were incubated for different time periods at 37 °C.

### Sperm viability assessment by Eosin Y staining

Briefly, 0.5 μl of 0.5% w/v Eosin Y solution was placed on a slide and 5 μl of sperm suspension was added. Immediately, the droplet was covered with a coverslip and the 100 sperm were examined under the microscope at a magnification of 40x (Plan-ACHROMAT, NA 0.65). Sperm heads stained with Eosin Y were classified as dead, while nonstained heads were considered alive sperm.

### Extraction of sperm proteins and immunoblotting

After incubation in the appropriate medium, sperm were washed by centrifugation (5 min at 720*g*), resuspended in sample buffer without reducing agents (62.5 mM Tris–HCl pH 6.8, 2% SDS, 10% glycerol) with phosphatase and protease inhibitors, and boiled for 5 min. After centrifugation for 5 min at 13500*g*, 5% β-mercaptoethanol and 0.0005% bromophenol blue was added to the supernatants, then boiled again for 5 min. Protein extracts equivalent to 5 to 6 × 10^6^ sperm per lane were separated by SDS-PAGE in gels containing either 8% (LIMK1 and SSH1), 12.5% (COFILIN), or 10% (PAK4) polyacrylamide. In all cases, proteins were transferred onto nitrocellulose membranes, and blots were blocked in 5% nonfat dry milk in PBS containing 0.1% Tween 20 (T-PBS) for 1 h at room temperature. An overnight incubation at 4 °C with the primary antibodies: anti-PAK4, anti-pLIMK1/2, anti-pSSH1, anti-pCOFILIN, anti-COFILIN, and anti-14-3-3 was required, while 1 h incubation at room temperature with the primary antibody anti-β-tubulin was sufficient. Antibodies were diluted in 2% nonfat dry milk in T-PBS as follows: 1:500 for anti-PAK4, anti-pLIMK1/2, and anti-pSSH1; 1:250 for anti-pCOFILIN, anti-COFILIN, and anti-14-3-3; 1:3000 for anti-β-tubulin. The corresponding secondary antibodies were incubated for 1 h at room temperature, diluted in 2% nonfat dry milk in T-PBS as follows: 1:3000 for HRP anti-rabbit and 1:3000 for HRP anti-mouse. In all cases, the reactive bands were visualized using a chemiluminescence detection solution consisting of 100 mM Tris-HCl buffer, pH 8, 0.205 mM coumaric acid, 1.3 mM luminol, and 0.01% H_2_O_2_ and were exposed for different time periods to CL-XPosure film (Thermo Scientific). Quantitative analysis was performed using ImageJ1.47 V software (National Institute of Health). The optical density of all bands was measured, relativized to β-tubulin, and then normalized to the respective control.

### Immunofluorescence

To perform immunofluorescence on mouse sperm, a previously described method was used (60). Briefly, after incubation in the appropriate medium, mouse sperm were washed twice by centrifugation for 5 min at 400*g* and then fixed with 4% paraformaldehyde in PBS (Sigma-Aldrich) for 10 min at room temperature. Sperm were washed twice by centrifugation for 5 min at 800*g*, resuspended in PBS, and placed onto glass slides. Air-dried sperm were washed twice for 5 min in T-PBS and then permeabilized with 0.5% Triton X-100 in PBS for 5 min. The slides were washed three times for 5 min in T-PBS and blocked in 3% BSA in PBS for 1 h at room temperature. After that, they were incubated with primary antibody (1:30 anti-PAK4 and 1:100 anti-pSSH1) diluted in PBS containing 1% BSA overnight at 4 °C. Following a washing step (three times, 5 min in T-PBS), slides were incubated with Alexa Fluor 568-conjugated anti-rabbit, diluted 1:200 in PBS containing 1% BSA for 1 h at room temperature, washed again for three times in T-PBS, and mounted using Vectashield mounting media (H-1000, Vector Labs). Nonspecific staining was determined by incubating the sperm in the absence of primary antibody.

For PNA staining, once the secondary antibody was washed, slides were incubated with FITC-conjugated PNA diluted 1:100 in PBS, for 1 h at room temperature. Slides were washed again three times in PBS and mounted using Vectashield mounting media.

In all cases, slides were examined using a fluorescence confocal microscope (Olympus IX83-DSU), and images were captured at 60x magnification (Plan Apo, NA 1.42 [oil]), with a sCMOS camera (Andor, Zyla).

### Fluorescence staining of actin filaments

After incubation in the appropriate medium, cells were fixed in 0.1% glutaraldehyde and 1.5% formaldehyde in PBS for 1 h and collected by centrifugation at 1300*g* for 5 min. The sperm pellet was immediately resuspended and incubated with 50 mM NH_4_Cl in PBS during 15 min and washed twice by resuspension/centrifugation in PBS and once in distilled water. Water-resuspended cells were used to prepare smears on glass slides, which were air-dried at room temperature. Sperm were rinsed with PBS for 7 min and then permeabilized using acetone at −20 °C for 7 min and washed three times in PBS. Slides were then incubated with TRITC-phalloidin (1:30) in PBS for 1 h at room temperature in humid conditions, in the dark. Sperm were washed three times with PBS, once in distilled water and air-dried at room temperature. Finally, they were mounted under coverglass slides using Vectashield mounting media. Nonspecific staining was determined by incubating the sperm in the absence of TRITC-phalloidin. Slides were examined using a fluorescence confocal microscope (Olympus IX83-DSU), and images were captured at 60x magnification (Plan Apo, NA = 1.42 [oil]), with a sCMOS camera (Andor, Zyla). The fluorescence intensity was quantified using ImageJ software1.47 V (National Institute of Health) and was calculated in regions of interest localized in the sperm head. The background intensity was subtracted.

### Acrosomal exocytosis

AR was assessed as previously described (41, 61). Briefly, mt-DsRed2 Acr-EGFP mouse sperm were resuspended to a final maximum concentration of 1 x 10^7^ cells/ml on NC medium. A 10-min preincubation in NC medium containing inhibitors was conducted when required. After that, sperm were incubated under capacitating conditions for 60 min in the presence or absence of inhibitors. At 60 min of incubation, progesterone (30 μM) was added for 30 min to stimulate the AR. Before collecting data, 2 ng/μl of propidium iodide was added to monitor viability. Data were recorded as individual cellular events using a BD FACSCanto II TM cytometer (Biosciences; Becton, Dickinson and Company). Side-scatter area and forward-scatter area data were collected from 20,000 events per sample to define sperm population. In all cases, doublet exclusion was performed analyzing two-dimensional dot plot forward-scatter area *versus* forward-scatter height. Positive cells for EGFP were collected using the filter for FITC (530/30) and for propidium iodide, the filter for peridinin chlorophyll protein complex (670LP). The two indicators had minimal emission overlap, but still compensation was done. Data were analyzed using FlowJo software (V10.0.7).

In one particular experiment, AR was assessed by Coomassie blue staining. Hybrid F1 mouse sperm were resuspended to a final maximum concentration of 1 x 10^7^ cells/ml on NC medium. A 10 min preincubation in NC medium containing inhibitors was conducted when required. After that, sperm were incubated under capacitating conditions for 60 min in the presence or absence of inhibitors. At 60 min of incubation, progesterone (30 μM) was added for 30 min to stimulate the AR. Sperm suspensions were fixed with 4% paraformaldehyde in PBS for 10 min at RT. Fixed sperm were washed three times with 100 mM ammonium acetate solution pH 9, by centrifugation at 500*g* for 5 min at RT, layered into microscope glass slides, and let them dry. Slides were washed in distilled water, cold methanol (−20 °C), and distilled water for 5 min each, submerged in Coomassie brilliant blue G-250 solution for 2 min, washed with distilled water, and mounted with 90% glycerol in PBS. Samples were immediately observed in a phase contrast light microscope with a 40x objective (Olympus CX31), and 400 cells were counted for negative or positive acrosome staining*.*

### Statistical analysis

Data are expressed as mean ± standard error of the mean of at least three independent experiments from different mice for all determinations. Statistical analysis was performed using the GraphPad Prism 6 software. Parametric or nonparametric comparisons were used as dictated by data distribution. The nonparametric Kruskal–Wallis test was performed in combination with Dunnˊs multiple comparisons test to analyze normalized data, such as optical density of WB bands (relativized to β-tubulin), or phalloidin fluorescence intensity. One-way ANOVA with Dunnett's multiple comparisons test was performed to analyze the percentage of sperm that undergo AR. For Coomassie blue data, arcsine transformation was performed before application of one-way ANOVA with Dunnett's multiple comparisons test. In some cases, Student *t* test was implemented to compare two specific conditions. One-way ANOVA with Tukey's multiple comparisons test was performed to analyze the percentage of sperm exhibiting each pSSH1 pattern. Finally, Chi-square test was used to analyze association between PAK4 and PNA patterns. A probability (p) value of *p* < 0.05 was considered statistically significant.

## Data availability

Data are to be shared upon request to the corresponding author.

## Supporting information

This article contains supporting information.

## Conflict of interest

The authors declare that they have no conflicts of interest with the contents of this article.

## Supporting information

Supplementary Figure 1Mouse sperm proteins (equivalent to 5 x 10^6^ sperm) were analyzed by 8-12.5% SDS-PAGE and immunoblotted using (*A*) anti-pLIMK1 (Thr908), (*B*) anti-COFILIN, or (*C*) anti-pCOFILIN (Ser3) antibodies

Supplementary Figure 2**Sperm viability assessment by Eosin Y staining.***A–C*, sperm were incubated under capacitating conditions for 60 min in the absence (V= DMSO) or presence of: PAK4 inhibitor PF-3758309 (PF), PP2A/PP1 inhibitor okadaic acid (OA), RAC1 inhibitor CAS 1177865-17-6 (CAS), SSH1 inhibitor sennoside A (SeA), RHOA/C inhibitor C3 transferase (C4), or PP2B inhibitor cyclosporin A (CsA). Immediately, sperm viability assessment by Eosin Y staining was performed by examination of 100 sperm per slide. One-way ANOVA was performed.
