# Supplementary figures and images for: The early molecular events leading to COFILIN phosphorylation during mouse sperm capacitation are essential for acrosomal exocytosis
Source: J Biol Chem. 2022 Apr 26;298(6):101988. doi: 10.1016/j.jbc.2022.101988 (PMC9142561; doi:10.1016/j.jbc.2022.101988)

## Supplementary Figure 1

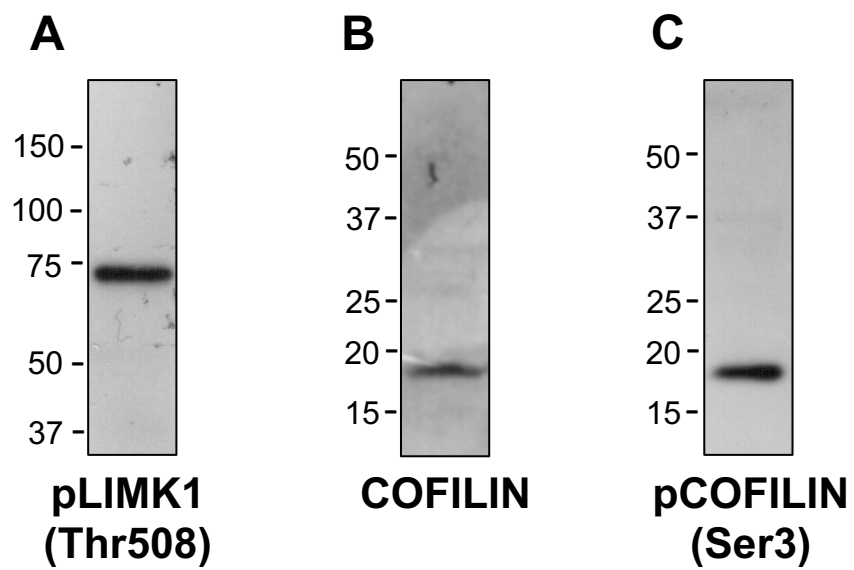

Supplement: Supplementary file 1 — Supplementary Figure 1Mouse sperm proteins (equivalent to 5 x 10^6^ sperm) were analyzed by 8-12.5% SDS-PAGE and immunoblotted using (A) anti-pLIMK1 (Thr908), (B) anti-COFILIN, or (C) anti-pCOFILIN (Ser3) antibodies [file mmc1.pdf]

# Supplementary Figure 2

A

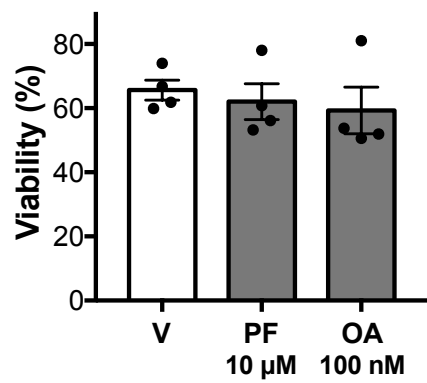

B

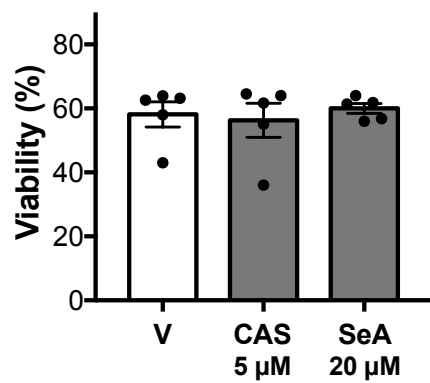

C

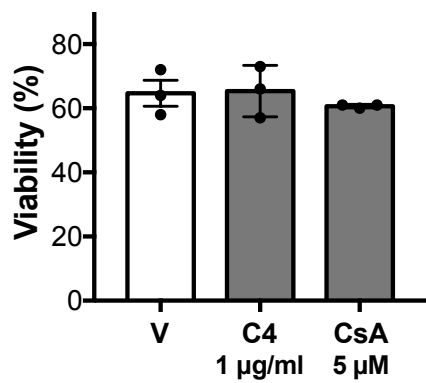

Supplement: Supplementary file 2 — Supplementary Figure 2Sperm viability assessment by Eosin Y staining.A–C, sperm were incubated under capacitating conditions for 60 min in the absence (V= DMSO) or presence of: PAK4 inhibitor PF-3758309 (PF), PP2A/PP1 inhibitor okadaic acid (OA), RAC1 inhibitor CAS 1177865-17-6 (CAS), SSH1 inhibitor sennoside A (SeA), RHOA/C inhibitor C3 transferase (C4), or PP2B inhibitor cyclosporin A (CsA). Immediately, sperm viability assessment by Eosin Y staining was performed by examination of 100 sperm per slide. One-way ANOVA was performed. [file mmc2.pdf]
